# The association between self-monitoring of blood glucose and HbA1c in type 2 diabetes

**DOI:** 10.3389/fendo.2023.1056828

**Published:** 2023-02-07

**Authors:** Yanping Yuan, Xianghai Zhou, Weiping Jia, Jian Zhou, Fan Zhang, Jianling Du, Linong Ji

**Affiliations:** ^1^ Department of Endocrinology, Peking University People’s Hospital, Beijing, China; ^2^ Department of Endocrinology & Metabolism, Shanghai Jiao Tong University Affiliated Sixth People’s Hospital, Shanghai Diabetes Institute, Shanghai, China; ^3^ Department of Endocrinology, Peking University Shenzhen Hospital, Shenzhen, Guangdong, China; ^4^ Department of Endocrinology and Metabolism, The First Affiliated Hospital of Dalian Medical University, Dalian, Liaoning, China

**Keywords:** type 2 diabetes, HbA1c, ridge regression, fasting capillary blood glucose, postprandial capillary blood glucose

## Abstract

**Aims:**

Fasting capillary blood glucose (FCG) and postprandial capillary blood glucose (PCG) both contribute to HbA1c in diabetes. Due to the collinearity between FCG and PCG, the HbA1c prediction model could not be developed with both FCG and PCG by linear regression. The study aimed to develop an HbA1c prediction model with both FCG and PCG to estimate HbA1c in type 2 diabetes.

**Methods:**

A total of 1,642 patients with type 2 diabetes who had at least three FCG and three PCG measurements in the past 3 months were enrolled in the study. The mean of FCG (MEAN_FCG_) and PCG (MEAN_PCG_) were calculated for each patient. The patients were randomized into exploratory and validation groups. The former was used for developing HbA1c prediction models and the latter for performance evaluation.

**Results:**

The new HbA1c prediction model using ridge regression expressed as HbA1c (%) = 0.320×MEAN_FCG_ (mmol/L) + 0.187×MEAN_PCG_ (mmol/L) + 2.979, R^2^ = 0.668. Compared to linear regression models developed with FCG, PCG, fasting plasma glucose (FPG), and 2-hour postprandial plasma glucose (2-h PPG), respectively, the new HbA1c prediction model showed the smallest mean square error, root mean square error, mean absolute error. The concordance correlation coefficient of the new HbA1c prediction model and the linear regression models with MEAN_FCG_, MEAN_PCG_, FPG or 2-h PPG were 0.810,0.773,0.749,0.715,0.672.

**Conclusion:**

We have developed a new HbA1c prediction model with both FCG and PCG, which showed better prediction ability and good agreement.

## Introduction

Glycated hemoglobin (HbA1c) is strongly correlated with mean blood glucose levels over 3 months. The American Diabetes Association (ADA) recommends that the goal of HbA1c is <7% in most non-pregnant adults with diabetes, which is directly associated with the reduction of diabetes complications (1). But HbA1c also has limitations in assessing blood glucose control. First, since HbA1c is measured once every 3 months and does not reflect the change in blood glucose promptly, HbA1c was not appropriate for assessing glycemic control in patients with adjusted hypoglycemic agents for less than 3 months. Second, there are marked discrepancies between blood glucose and HbA1c level for patients such as hemoglobin variant, thalassemia, hemolysis, recent blood transfusion and pregnancy (2). For those who are not suitable for HbA1c mentioned above, continuous glucose monitoring (CGM) and self-monitoring of blood glucose (SMBG) provide immediate spot glucose readings in diabetes. CGM is expensive and not available for all type 2 diabetes, while SMBG is undoubtedly a convenient and cost-effective method of blood glucose monitoring that is widely used by patients with diabetes to guide the timely modification of diabetes treatment regimens (3).

Studies on the association between SMBG and HbA1c were limited in patients with type 2 diabetes. The A1c-Derived Average Glucose (ADAG) study provided the correlation between HbA1c and mean blood glucose from CGM and SMBG in patients with type 1 diabetes, type 2 diabetes and nondiabetic, and allowed the calculation of estimated mean blood glucose for a given HbA1c (4). However, the correlation in ADAG was not appropriate for type 2 diabetes with less frequent blood glucose monitoring that was not sufficient to obtain average blood glucose. Compared with premeal glucose or postprandial glucose, the immediate clinical value of mean blood glucose in day-to-day blood glucose monitoring and treatment was limited. Although previous studies have reported the relationship between fasting capillary blood glucose (FCG) or postprandial capillary blood glucose (PCG) with HbA1c (5, 6), there is no doubt that both FCG and PCG contribute to HbA1c levels (5), so the prediction of HbA1c using FCG or PCG alone may be inaccurate. In clinical practice, it can be observed that some patients only reach the FCG target or the PCG target, how to predict the HbA1c levels of these patients individually is unknown for now. Therefore, it is necessary to take both FCG and PCG into account when analyzing the relationship between blood glucose and HbA1c. In the present study, we analyzed the association between FCG, PCG, and HbA1c using data from Chinese patients with type 2 diabetes who had stable diabetes treatment, and thus develop a new HbA1c prediction model based on both FCG and PCG.

## Materials and methods

### Study population

The multi-center, observational study enrolled patients with type 2 diabetes in endocrinology departments of eight hospitals in China from March 2018 to Jan 2020. The inclusion criteria included the following: 1) Type 2 diabetes aged ≥18 years referred to the endocrinology department; 2) being untreated, or receiving stable antidiabetic treatment including diet, exercise, or hypoglycemic agents for at least 3 months before the study; 3) having at least three FCG and three PCG measurements over a 3-month period before the study. The exclusion criteria included the following: 1) being pregnant or lactated; 2) using drugs that elevate blood glucose such as glucocorticoids, chemotherapy drugs within 3 months; 3) having conditions that may change blood glucose such as infection, myocardial infarction, tumor, inflammation, trauma, hyperthyroidism, hypothyroidism, Cushing’s syndrome or acromegaly within 3 months; 4) having a history of severe liver disease or alanine transaminase/aspartate transaminase ≥3 times the normal upper limit; 5) having a history of serious kidney disease or serum creatinine >133 μmol/L; 6) having known hematological disease or hemoglobin <90 g/L; 7) being hypoproteinemia with serum albumin <35g/L; 8) blood transfusion or blood donation within 3 months. The Ethics Committee of the Peking University People’s Hospital approved the study protocol. All patients signed informed consent before the interview and data collection. In this study, data were collected from 1737 patients with type 2 diabetes. After excluding 24 patients diagnosed with diabetes for less than 3 months, 5 patients who were found to have hemoglobin variants, 11 patients with hemoglobin <90 g/L, 16 patients with serum creatinine >133 umol/L, 22 patients with serum albumin <35 g/L, 9 patients with alanine transaminase/aspartate transaminase ≥3 times the normal upper limit, and 8 patients with glycated albumin (GA) information missing, data from 1642 patients were used for data analysis. The patients were randomized into an exploratory group including 819 patients and a validation group including 823 patients.

### Data collection

Demographic characteristics and medical history of the patients were recorded. Patients’ glucose-lowering medications, records of 3 FCGs and 3 to 9 PCGs in SMBG, smoking, and alcohol consumption during the last 3 months before the interview were collected. Body weight and height were measured using a calibrated scale and body mass index (BMI, kg/m^2^) was calculated. Blood pressure was measured using a mercury sphygmomanometer.

### Laboratory assessments

Venous blood samples were drawn in the morning after an overnight fast. Fasting plasma glucose (FPG), HbA1c, hemoglobin, serum creatinine, albumin, alanine transaminase, aspartate transaminase, total cholesterol, and triglycerides were measured in local laboratories. Patients had breakfast and take their daily hypoglycemic agents. The blood sample was collected 2 hours later to measure 2-hour postprandial plasma glucose (2-h PPG). Glycated hemoglobin HbA1c was tested using ion-exchange high-performance liquid chromatography, capillary electrophoresis, immunoassay, enzymatic assay or boronate affinity chromatography in local laboratories. Local laboratories were required to perform 10 samples comparison quarterly with the Department of Laboratory Medicine, Zhongshan Hospital, Fudan University, which is an HbA1c Secondary Referral Laboratory certificated by the National Glycohemoglobin Standardization Program (NGSP) (7). At 1 year, all local laboratories achieved a deviation of ≤6% in at least 38 of 40 HbA1c results. Serum and whole blood specimens were stored at -80°C and transported to the central laboratory at Peking University People’s Hospital for GA and hemoglobin electrophoresis. GA was measured by enzymatic methods (Lucica GA‐L, Japan). Hemoglobin electrophoresis was performed to screen the Hb variant using the method of capillary electrophoresis (Minicap Flex Piercing, Sebia, France).

### Definition

FCG referred to the capillary blood glucose before breakfast of a day. PCG referred to the capillary blood glucose 2 hours after breakfast, lunch or dinner. The mean of FCG (MEAN_FCG_) and PCG (MEAN_PCG_) were calculated for each patient. The MEAN_PCG_ was the mean PCG of each patient after three meals. The mean PCG of breakfast (MEAN_PCGB_), lunch (MEAN_PCGL_) and dinner (MEAN_PCGD_) were also calculated for each patient.

Criteria for the diagnosis of type 2 diabetes and definition of HbA1c goal achievement of <7% were based on American Diabetes Association ADA (2022) guidelines (1). FCG control target range was 80-130 mg/dL (4.4-7.2 mmol/L) according to ADA guideline and 80-126 mg/dL (4.4-7.0 mmol/L) according to the Chinese Diabetes Society (CDS) (8). The target of PCG control was <180mg/dL (10.0 mmol/L) according to the ADA and CDS guidelines. Medical history of type 2 diabetes was also an auxiliary criterion for the diagnosis of type 2 diabetes. Hypoglycemia was defined as the patient’s SMBG recorded blood glucose or plasma glucose concentration <3.9 mmol/L, as well as self-reported hypoglycemic symptoms.

### Statistical analysis

Data analysis was performed using SPSS software (version 23.0; IBM Corp., Armonk, New York, USA). Continuous variables were presented as mean ± standard deviation or median (interquartile range). Categorical variables were expressed as number (percentage).

The patients were randomized into exploratory and validation groups by SPSS software. HbA1c prediction models were developed in the exploratory group and validated in the validation group. The correlation coefficient between MEAN_FCG_ and MEAN_PCG_ was 0.827 (P <0.001). Correlation coefficient >0.8 was considered collinearity (9). Due to the collinearity between MEAN_FCG_ and MEAN_PCG_, the ridge regression model was used to develop a new HbA1c prediction model using both MEAN_FCG_ and MEAN_PCG_ to predict HbA1c. Ridge parameter k (lambda) was introduced to the regression equation to make the estimated value of the regression coefficient essentially stable. The trend of ridge trace became stable with the optimal ridge parameter k (10). The optimal value of ridge parameter k was selected by the machine learning method with cross-validation using Python 3.8. Simple linear regression models were developed to predict HbA1c with MEAN_FCG_, MEAN_PCG_, FPG, and 2-h PPG, respectively. The difference in performance between the new HbA1c prediction model and simple linear regression models was assessed by mean square error (MSE), root mean square error (RMSE), mean absolute error (MAE), and coefficient of determination (R^2^) in the validation group. The MSE, RMSE, MAE was calculated by


$$MSE=\frac{1}{m}\sum_{i=1}^{m}{\left(X_{i}-Y_{i}\right)}^{2},\;RMSE=\sqrt{\frac{1}{m}\sum_{i=1}^{m}{\left(X_{i}-Y_{i}\right)}^{2}},\;MAE=\frac{1}{m}\sum_{i=1}^{m}\left|X_{i}-Y_{i}\right|$$

respectively. *X_i_* represents the predicted HbA1c, *Y_i_* represents the actual measured HbA1c, m means the sum of participants. The smaller MSE, RMSE, and MAE demonstrated the better accuracy of the prediction model (11).

Bland–Altman plots and concordance correlation coefficient (CCC) were used to evaluate the agreement between actual HbA1c and predicted HbA1c in the validation group. The CCC >0.80 suggested a strong agreement between actual and predicted values. Receiver operating characteristic (ROC) curves were used to assess the sensitivity and specificity of the new HbA1c prediction model and simple linear regression models to detect patients with HbA1c <7% using MedCalc version 20.0. The areas under the ROC curves (AUC) of HbA1c prediction models were calculated and compared using the DeLong test. All *P*-values were two-tailed and *P*-values <0.05 were considered statistically significant.

## Results

### Patient characteristics

There were 1642 patients with a mean age of 59.3 ± 11.0 years in the study. Males accounted for 61.9%. The median duration of type 2 diabetes was 7.9 years. Patients had an average MEAN_FCG_ of 8.29 ± 2.64 mmol/L, average MEAN_PCG_ of 11.55 ± 3.73 mmol/L, and mean HbA1c of 7.80 ± 1.86%. The mean number of daily glucose tests was 7.51 ± 2.08. The percentage of patients experiencing hypoglycemia was 11.1% within 3 months. The clinical characteristics of the patients were not statistically different between the exploratory group and the validation group (**Table 1**). The mean number of daily glucose tests was not significantly different between those on non-insulin-treated and insulin-treated patients in the exploratory group (7.49 ± 2.06 vs 7.45 ± 2.00, *P*=0.784), but the mean number of daily glucose tests in non-insulin-treated patients was more than those in insulin treatment in the validation group (7.68 ± 2.20 vs 7.28 ± 1.95, *P*=0.011).

**Table 1:** Characteristics of 1642 patients with type 2 diabetes.

Variable	Total population	Exploratory group	Validation group
Subjects, n	1642	819	823
Male, n (%)	1017 (61.9)	507 (61.9)	510 (62.0)
Smoking^†^, n (%)	389 (23.7)	188 (23.0)	201 (24.4)
Drinking^†^, n (%)	241 (14.7)	116 (14.2)	125 (15.2)
Age, years	59.3 ± 11.0	59.2 ± 11.2	59.4 ± 10.7
Duration of diabetes, years	7.9 (2.6, 14.3)	8.0 (2.6, 14.5)	7.7 (2.5, 14.0)
BMI, kg/m^2^	25.45 ± 3.46	25.47 ± 3.48	25.42 ± 3.43
SBP, mmHg	132.81 ± 17.19	132.39 ± 16.85	133.23 ± 17.51
DBP, mmHg	79.86 ± 10.27	79.56 ± 10.12	80.16 ± 10.41
Total cholesterol, mmol/L	4.48 ± 1.27	4.49 ± 1.18	4.48 ± 1.36
Triglyceride, mmol/L	1.53 (1.08, 2.26)	1.59 (1.12, 2.33)	1.46 (1.05, 2.16)
FPG, mmol/L	8.57 ± 3.25	8.54 ± 3.24	8.60 ± 3.25
2-h PPG, mmol/L	12.86 ± 5.12	12.86 ± 5.23	12.84 ± 5.00
HbA1c, %	7.80 ± 1.86	7.82 ± 1.87	7.79 ± 1.85
GA, %	20.89 ± 6.37	20.83 ± 6.24	20.95 ± 6.51
MEAN_FCG_, mmol/L	8.29 ± 2.64	8.30 ± 2.66	8.28 ± 2.62
MEAN_PCG_, mmol/L	11.55 ± 3.73	11.66 ± 3.85	11.44 ± 3.61
Number of daily glucose tests	7.51 ± 2.08	7.47 ± 2.04	7.54 ± 2.13
Diabetes treatment, n (%)			
OAD	1175 (71.6)	596 (72.8)	579 (70.4)
One OAD	550 (46.8)	292 (49.0)	258 (44.6)
Two OADs	468 (39.8)	219 (36.7)	249 (43.0)
≥Three OADs	157 (13.4)	85 (14.3)	72 (12.4)
Insulin, n (%)	585 (35.6)	305 (37.2)	280 (34.0)
Hypoglycemia*^†^, n (%)	191 (11.6)	95 (11.6)	96 (11.7)

Data are shown as mean ± standard deviation, median (interquartile range), or n (%).

BMI, body mass index; SBP, systolic blood pressure; DBP, diastolic blood pressure; FPG, fasting plasma glucose; 2-h PPG, 2-hour postprandial plasma glucose; HbA1c, glycated hemoglobin; GA, glycated albumin; MEAN_FCG_, average fasting capillary glucose (FCG) for each patient; MEAN_PCG_, average postprandial capillary glucose (PCG) for each patient; OAD, oral anti-hyperglycemic drug.

* Hypoglycemia was defined as the patient’s SMBG recorded blood glucose or plasma glucose concentration < 3.9mmol/L, as well as self-reported hypoglycemia symptom.

† within 3 months before the study.

### HbA1c prediction models and their performance

**Figure 1:**
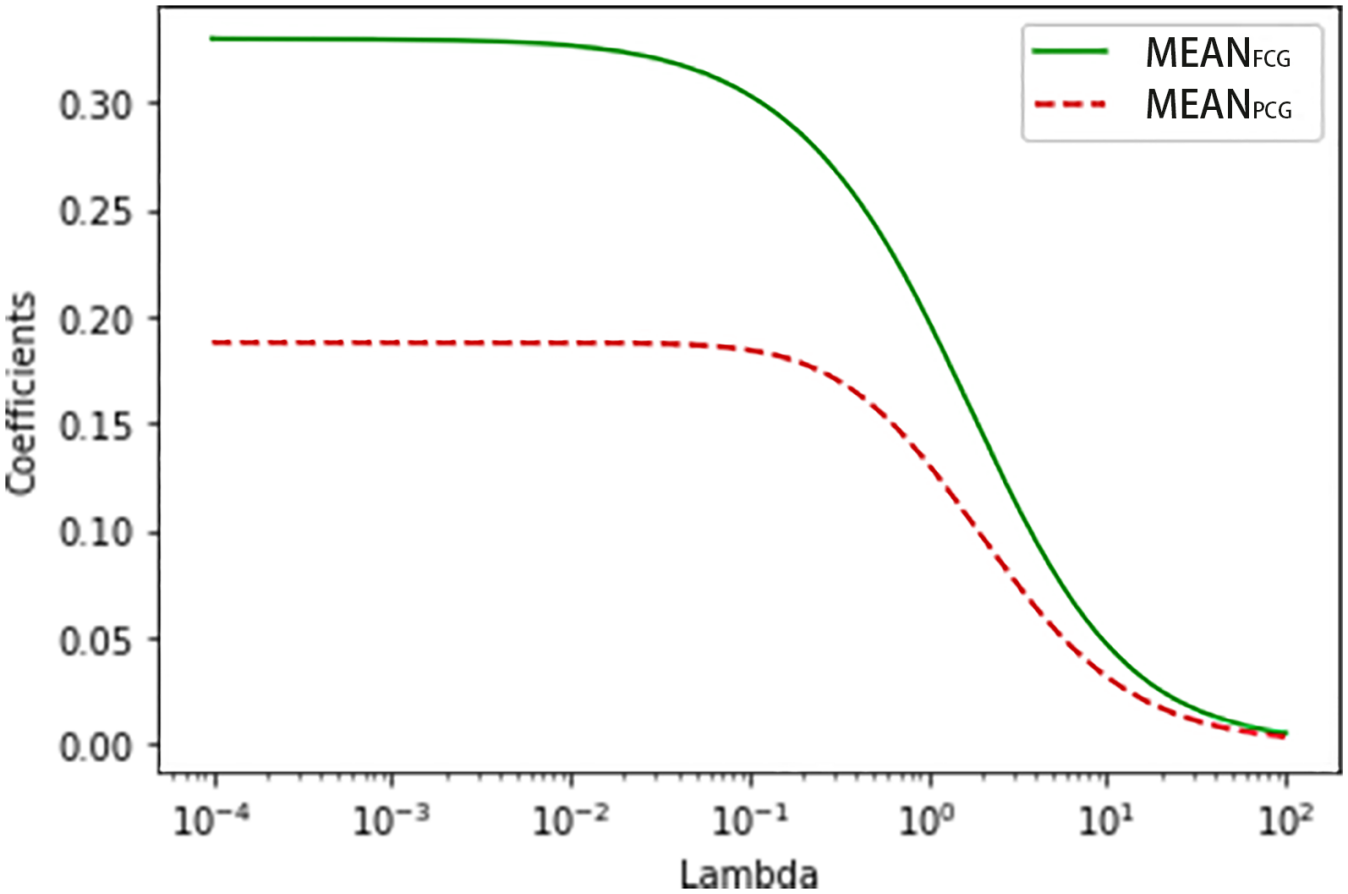
The ridge trace of the ridge regression model for predicting HbA1c with MEAN_FCG_ and MEAN_PCG_.

In the exploratory group, the trend of ridge trace became stable when ridge parameter k was 0.03 (**Figure 1**). The new HbA1c prediction model expressed as HbA1c (%) = 0.320×MEAN_FCG_ (mmol/L) + 0.187×MEAN_PCG_ (mmol/L) + 2.979 when k = 0.03. The four simple linear regression models of HbA1c based on MEAN_FCG_, MEAN_PCG_, FPG, or 2-h PPG expressed as HbA1c (%) = 0.554×MEAN_FCG_ (mmol/L) +3.218, HbA1c (%) = 0.376×MEAN_PCG_ (mmol/L) +3.434, HbA1c (%) = 0.417×FPG (mmol/L) +4.251 and HbA1c (%) = 0.256×(2-h PPG) (mmol/L) +4.527, respectively. The R^2^ of the new HbA1c prediction model and the linear regression model with MEAN_FCG_, MEAN_PCG_, FPG or 2-h PPG was 0.668, 0.622, 0.599, 0.523 and 0.511, respectively, indicating that the predictors of the new HbA1c prediction model were more strongly correlated with HbA1c compared with the other linear regression models (**Table 2**). In addition, the ridge regression model of HbA1c based on both FPG and 2-h PPG expressed as HbA1c (%) = 0.248×FPG (mmol/L) + 0.144×(2-h PPG) (mmol/L) + 3.844 when k = 0.02, R^2 =^ 0.639. Sensitivity analysis of PCG was performed. In patients who had PCG of three meals (N=417), the R^2^ of linear regression model of HbA1c based on MEAN_PCGB_, MEAN_PCGL_ and MEAN_PCGD_ was 0.556, 0.504 and 0.506, respectively. And R^2^ of the linear regression model of 2-h PPG and HbA1c in 417 patients was 0.496.

**Table 2:** The comparison of ridge regression model and simple linear regression model for HbA1c.

Model	Variable	R^2^	MSE	RMSE	MAE	Mean difference	95%LoA	PA, %	CCC
Ridge regression	MEAN_FCG_, MEAN_PCG_	0.668	1.038	1.019	0.742	0.03	(-1.97,2.02)	93.68	0.810 (0.787, 0.830)
Linear regression	MEAN_FCG_	0.622	1.253	1.120	0.826	-0.01	(-2.21,2.18)	93.92	0.773 (0.746, 0.797)
	MEAN_PCG_	0.599	1.318	1.148	0.852	0.06	(-2.19,2.30)	93.20	0.749 (0.721, 0.775)
	FPG	0.523	1.498	1.224	0.912	-0.04	(-2.44,2.36)	93.92	0.715 (0.683, 0.744)
	2-h PPG	0.511	1.659	1.288	0.959	-0.02	(-2.55,2.50)	95.02	0.672 (0.637, 0.703)

R^2^, coefficient of determination; MSE, mean square error; RMSE, root mean square error; MAE, mean absolute error; 95% limits of agreement, 95% LoA; PA, percentage of agreement; CCC, concordance correlation coefficient.

**Figure 2:**
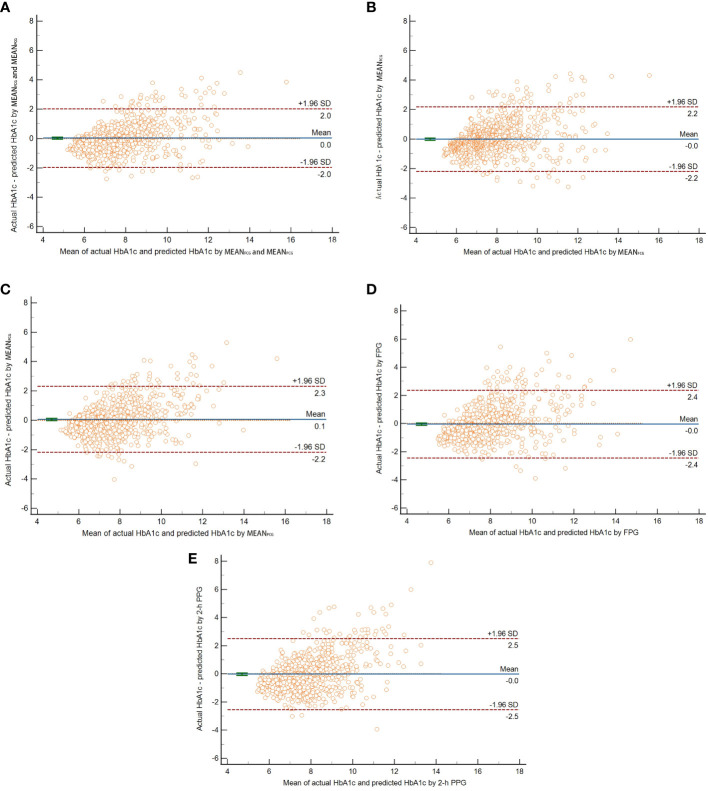
The Bland-Altman plot of actual HbA1c and predicted HbA1c by ridge regression with both MEAN_FCG_ and MEAN_PCG_ **(A)**, linear regression with MEAN_FCG_ **(B)**, MEAN_PCG_ **(C)**, FPG **(D)**, or 2-h PPG **(E)** respectively. The horizontal solid line in the middle indicated the mean difference between actual HbA1c and predicted HbA1c. The upper and lower horizontal dotted lines indicated the 95% limits of agreement.

In the validation group, the new HbA1c prediction model yielded the smallest value of MSE, RMSE, and MAE compared with four simple linear regression models with MEAN_FCG_, MEAN_PCG_, FPG, or 2-h PPG in the validation group (**Table 2**). The Bland-Altman plot showed that the mean difference (95% limits of agreement) was 0.03(-1.97,2.02) between actual HbA1c and predicted HbA1c with the new prediction model (**Figure 2**). The mean difference of four simple linear regression models by MEAN_FCG_, MEAN_PCG_, FPG, or 2-h PPG was also close to zero, with -0.01(-2.21,2.18), 0.06(-2.19,2.30), -0.04(-2.44,2.36), -0.02(-2.55,2.50), respectively. Most of the differences between actual HbA1c and predicted HbA1c in different prediction models were within 95% limits of agreement. Of the new HbA1c prediction model and simple linear regression models, only the CCC of the new HbA1c prediction model was greater than 0.80, indicating that the predicted HbA1c with the new HbA1c prediction model had a stronger agreement with actual HbA1c (**Table 2**). After removing patients who experienced hypoglycemia, the HbA1c prediction models showed similar performance (the results were not shown here).

### The ability of HbA1c prediction models to detect patients with HbA1c <7%

**Figure 3:**
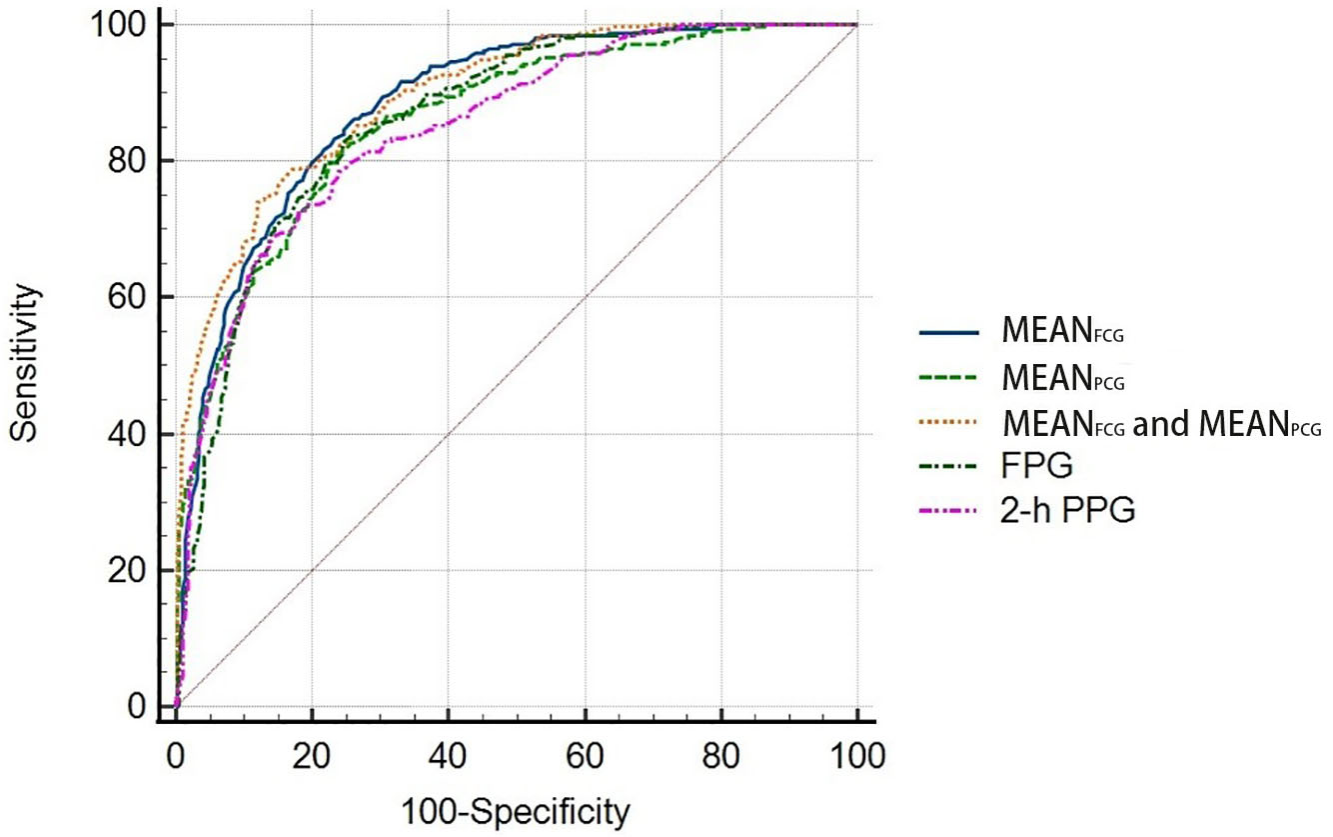
Receiver operating characteristic (ROC) curves for the HbA1c prediction models to detect patients with HbA1c <7%.

In the validation group, the new HbA1c prediction model for predicting HbA1c with MEAN_FCG_ and MEAN_PCG_ showed strong predictive ability to detect patients who had HbA1c <7% with AUC of 0.895 (95% CI: 0.872, 0.915), which was higher than simple linear regression models with MEAN_PCG_ [0.860 (0.835, 0.883), *P *<0.0001], FPG [0.863 (0.838, 0.886), *P*=0.011] or 2-h PPG [0.850 (0.824, 0.874), *P*=0.001]. But the new HbA1c prediction model did not show better predictive ability compared to the simple linear regression model with MEAN_FCG_ [0.884 (0.860, 0.905), *P*=0.107] (**Figure 3**).

### Consistency of actual and predicted HbA1c grouped with FCG and PCG control targets

Patients were grouped with MEAN_FCG_ ≤7.2 and >7.2 mmol/L, MEAN_PCG_ <10.0 and ≥10.0 mmol/L, respectively. More than 80% of patients had the consistency of actual and predicted HbA1c for both <7% and both ≥7% in patients with both MEAN_FCG_ and MEAN_PCG_ within/outside the control target range (**Table 3**). In patients who had MEAN_FCG_ within the control target range and MEAN_PCG_ ≥10.0 mmol/L, a proportion of 35.1% patients had actual HbA1c <7% but predicted HbA1c ≥7%. And two of these patients with actual HbA1c <7% but predicted HbA1c ≥7% had experienced hypoglycemia, one of them was given insulin treatment and the other one was given insulin secretagogues, compared with no one had experienced hypoglycemia in both actual and predicted HbA1c <7%. In patients who had MEAN_FCG_ >7.2 mmol/L and MEAN_PCG_ within the control target range, patients with actual HbA1c <7% but predicted HbA1c ≥7% accounted for 20.6%. Only one patient with insulin use had experienced hypoglycemia compared with no one who had experienced hypoglycemia in both actual and predicted HbA1c <7%.

**Table 3:** Actual and predicted HbA1c grouped with FCG and PCG control targets.

Group	actual <7% predicted <7%	actual <7% predicted ≥7%	actual ≥7% predicted <7%	actual ≥7% predicted ≥7%
ADA guideline				
MEAN_FCG_ ≤ 7.2mmol/L				
MEAN_PCG_ <10.0mmol/L	192 (79.7)	9 (3.7)	35 (14.5)	5 (2.1)
MEAN_PCG_ ≥10.0mmol/L	6 (6.4)	33 (35.1)	9 (9.6)	46 (48.9)
MEAN_FCG_ >7.2mmol/L				
MEAN_PCG_ <10.0mmol/L	7 (9.6)	15 (20.6)	6 (8.2)	45 (61.6)
MEAN_PCG_ ≥10.0mmol/L	0	49 (11.8)	0	366 (88.2)
CDS guideline				
MEAN_FCG_ ≤ 7.0mmol/L				
MEAN_PCG_ <10.0mmol/L	190 (84.4)	31.2	31 (13.8)	1 (0.4)
MEAN_PCG_ ≥10.0mmol/L	6 (8.7)	22 (31.2)	9 (13.0)	32 (46.4)
MEAN_FCG_ >7.0mmol/L				
MEAN_PCG_ <10.0mmol/L	9 (10.1)	21 (23.6)	10 (11.2)	49 (55.1)
MEAN_PCG_ ≥10.0mmol/L	0	60 (13.6)	0	380 (86.4)

Data are shown as n (%).

Similar results were found when patients were grouped with MEAN_FCG_ ≤7.0 and >7.0 mmol/L, MEAN_PCG_ <10.0 and ≥10.0 mmol/L, respectively.

## Discussion

Our study analyzed the correlation of MEAN_FCG_ and MEAN_PCG_ with HbA1c using ridge regression and developed a new model for predicting HbA1c by combining MEAN_FCG_ and MEAN_PCG_. The new HbA1c prediction model predicted HbA1c with better performance than the HbA1c prediction model using MEAN_FCG_, MEAN_PCG_, FPG, or 2-h PPG alone. The new HbA1c prediction model had a better predictive ability to detect patients who had HbA1c <7% than simple linear model with MEAN_PCG_, FPG, or 2-h PPG, but was similar to the simple linear model with MEAN_FCG_.

To our knowledge, our study was the first to use a ridge regression model to establish a model using FCG and PCG together to predict HbA1c in patients with type 2 diabetes. This new HbA1c prediction model was appropriate for patients who were not suitable for HbA1c measurement after adjusting hypoglycemic treatment for less than 3 months, especially when patients only reached FCG or PCG target. Individualized predicted HbA1c with SMBG results was used to determine whether the hypoglycemic treatment should be adjusted to promote HbA1c to reach the goal quickly. When the patient’s actual HbA1c was inconsistent with the predicted HbA1c, the cause should be actively sought, such as frequent hypoglycemia, anemia, hemolysis, pregnancy, hemoglobinopathy, etc. In these patients, the new HbA1c prediction model might be applied to evaluate long-term glycemic control.

Our study showed that FCG had a stronger predictive performance for HbA1c than FPG alone, which was similar to the results of a previous study (5). When comparing PCG after three meals and 2-h PPG, it seems that PCG after breakfast was better than that after lunch, after dinner, and 2-h PPG, indicating that PCG after breakfast was more important than other meals, and superior to intravenous blood glucose after breakfast. This suggested that patients should pay more attention to PCG after breakfast in the PCG after three meals. Moreover, the R^2^ of the model developed using both FPG and 2-h PPG was lower than that of the new HbA1c prediction model using both MEAN_FCG_ and MEAN_PCG_, indicating that SMBG at home was better than single intravenous blood glucose measurement in hospital when predicting HbA1c. A possible explanation was that FPG and 2-h PPG had day-to-day variability (12–14), so that the average blood glucose level over a period of time was a more accurate reflection of HbA1c levels than a single plasma glucose test.

Compared with the new prediction model, simple linear model with FCG showed comparable ability to identify patients with HbA1c <7%, but the simple linear model with PCG showed worse ability. It indicated that FCG had a higher value than PCG in determining whether patients had achieved an HbA1c goal of <7%. This was consistent with previous reports that premeal blood glucose was more closely related to HbA1c than postmeal blood glucose (15). In patients without monitoring PCG, FCG alone can be used to identify whether a patient is meeting the HbA1c goal using the simple linear model with FCG.

There was a discrepancy between the actual HbA1c and predicted HbA1c by the new HbA1c prediction model in this study. In patients who had only FCG within control target range, about 1/3 of patients with actual HbA1c <7.0% had predicted HbA1c ≥7%. Compared to patients with actual and predicted HbA1c <7.0%, we found that more patients had experience hypoglycemia in those with actual HbA1c <7.0% and predicted HbA1c ≥7%. The possible explanation was that these patients had a higher risk of hypoglycemia, which lead to the actual HbA1c reaching the goal. At this time, the actual HbA1c might be inaccurate, suggesting that clinicians should not reduce blood glucose excessively to further increase the risk of hypoglycemia in this case. For those who had only PCG within control target range, 1/4 of the patients with actual HbA1c <7% had predicted HbA1c ≥7%. Limited by the number of people with hypoglycemia occurrence, it was difficult to speculate whether these people had an increased risk of hypoglycemia.

Studies have found that blood glucose testing number correlated with HbA1c attainment in insulin-treated patients (16, 17). In our study, although the frequency of blood glucose was different in insulin-treated patients, the average blood glucose was finally included in the model for HbA1c prediction. In addition, there was no difference in the number of blood glucose tests between the insulin-treated and non-insulin-treated patients in the model-established group, which could avoid the interference to the prediction model caused by the increased number of tests in insulin-treated patients. Although more glucose tests were observed in non-insulin-treated patients than insulin-treated patients in the validation group, studies showed that SMBG frequency was not associated with glycemic control in non-insulin-treated patients (18, 19).

The strength of this study was that it was a multicenter study, and only required type 2 diabetes with insulin-treated and non-insulin-treated to have at least three FCG and three PCG measurements, indicating that this study had good extrapolation in type 2 diabetes. Several limitations exist in the current study. First, SMBG measurements were performed by the patient’s glucose-monitoring devices. Improper operation during blood glucose measurement, expiration of the test strip, and failure to calibrate might affect the accuracy and precision of the glucose readings. Second, the SMBG values were collected from four points including fasting and after three meals for several days over 3 months, some higher or lower values might be missed. But each point glucose value had at least 3 measurements for each patient with stable hypoglycemic treatment, which avoid a snapshot of glucose variability and reduce the impact of intra-individual differences. Third, validation of the new HbA1c prediction model was performed internally, and further validation of the model in external populations will be required in the future. Fourth, the efficacy and safety of this new HbA1c prediction model remained to be investigated, and we are conducting clinical trials to evaluate its efficacy and safety in patients with type 2 diabetes who have just been adjusted for hypoglycemic treatment.

In conclusion, we have established the association between FCG, PCG and HbA1c and developed a new HbA1c prediction model based on both FCG and PCG. The new HbA1c prediction model provided an available and convenient way to convert real-time SMBG readings to HbA1c. Applying the new HbA1c prediction model might help to make the most of SMBG information and promote HbA1c to reach the goal quickly in patients with type 2 diabetes.

## Data availability statement

The original contributions presented in the study are included in the article/supplementary material. Further inquiries can be directed to the corresponding authors.

## Ethics statement

The studies involving human participants were reviewed and approved by Peking University People’s Hospital. The patients/participants provided their written informed consent to participate in this study.

## Author contributions

XZ designed this study. YY drafted the initial manuscript. XZ and LJ revised the manuscript. XZ, WJ, JZ, FZ, JD were responsible for collecting and managing the data of their hosipital. All authors contributed to the article and approved the submitted version.
